# Online Distributed User Association for Heterogeneous Radio Access Network

**DOI:** 10.3390/s19061412

**Published:** 2019-03-22

**Authors:** B. Bikram Kumar, Lokesh Sharma, Shih-Lin Wu

**Affiliations:** 1 Department of Electrical Engineering, Chang Gung University, Taoyuan 33302, Taiwan; basaba.bikram@gmail.com; 2 Department of AI Innovation Research Center, Chang Gung University, Taoyuan 33302, Taiwan; engglucky@gmail.com; 3 Department of Computer Science and Information Engineering, Chang Gung University, Taoyuan 33302, Taiwan; 4 Department of Cardiology, Chang Gung Memorial Hospital, Taoyuan 33305, Taiwan; 5 Department of Electrical Engineering, Ming Chi University of Technology, New Taipei City 24301, Taiwan

**Keywords:** load balancing, online user association, HetNet, distributed scheme

## Abstract

Future-generation radio access networks (RAN) are projected to fulfill the diverse requirements of user equipment (UE) by adopting a heterogeneous network (HetNet) environment. Necessary integration of different radio access technologies (RAT), such as 2G, 3G, 4G, wireless local area network (WLAN), and visible light communication (VLC) is inevitable. Moreover, UEs equipped with diverse requirements will be capable of accessing some or all the RATs. The complex HetNet environment with diverse requirements of UEs will present many challenges. The HetNet is likely to suffer severely from load imbalance among the base stations (BSs) from inheriting the traditional user association scheme such as max-SINR (signal-to-interference-plus-noise ratio)/max-RSSI (received signal strength indicator), unless some sophisticated schemes are invented. In this paper, a novel scheme is devised for a joint-user association for load balancing, where BSs are densely deployed and UEs typically have a certain degree of mobility. Unlike most of the present works, a dynamic network is considered where the position and channel condition of the UEs are not fixed. We develop two complex and distributed association schemes based on *probability* and *d-choices*, while carefully considering both loads of the BSs and SINR experienced by the UEs. Numerical results validate the efficiency of the proposed schemes by showing a received data-rate fairness among UEs and an improvement in the UE’s minimum received data rate.

## 1. Introduction

**Figure 1:**
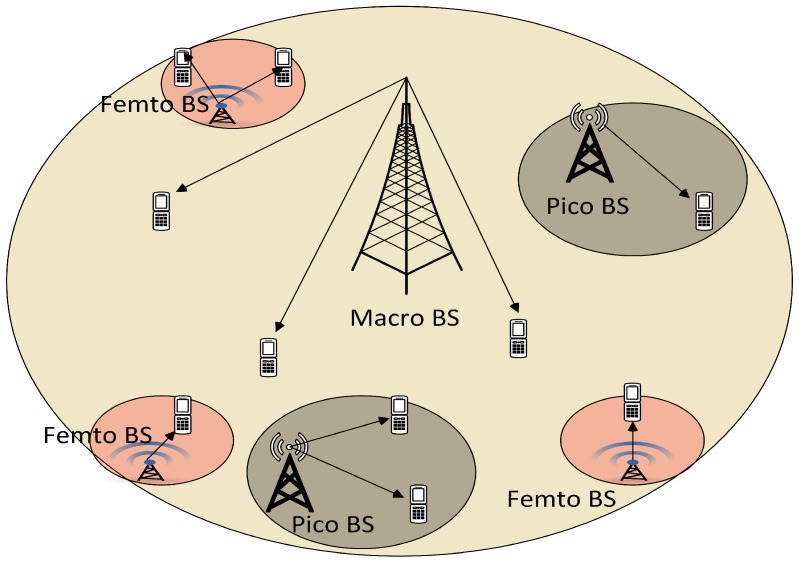
Heterogeneous radio access network (RAN) system architecture.

The traffic demand in wireless communication systems has surged over the past few years. This trend will continue in the future because of the growing popularity and heavy usage of wireless devices [1]. According to telecom players [2], the average mobile network connection speed globally will increase three-fold from 2016 (6.8 Mbps) to 2021 (20.3 Mbps). By 2021, the average global mobile connection speed will surpass 20 Mbps, there will be 1.5 mobile devices per capita, and smart-phones will cross 86% of mobile data traffic. All these developments will lead to an outburst of wireless traffic volume on limited spectrum resources. To meet these surging traffic demands, the requirement of a sophisticated radio access network (RAN) integrated with multiple radio access technologies (RAT) is inevitable. Heterogeneous RAN is proposed to solve the above traffic demand. This heterogeneous network (HetNet) will be an integration of different technologies, such as 2G, 3G, 4G, wireless local area network (WLAN), visible light communication (VLC) [3,4], mobile device cloud (MDC) [5], and vehicular cloud computing [6]. This is to ensure backward compatibility, as well as to support current and future technologies [7]. This HetNet model can support different applications, such as the multi-cloud service [8], data aggregation [9,10], and monitoring of critical systems [11]. It has been widely accepted that HetNet has the potential to achieve high spectral and energy efficiency. Ease of deployment and maintenance is an added advantage of HetNet deployment. In fact, to enhance traffic volume, 3rd Generation Partnership Project (3GPP) long-term evolution advanced (LTE-A) proposes a multi-tier HetNet, in which low-powered and short-range small cells/tiers (picocells, femtocells) are laid under macro base stations (BSs) to fulfill Quality of service (QoS) requirements and traffic volume [12], as shown in Figure 1. The densification with different kinds of BSs in HetNet creates a huge problem of load imbalance in the networks. In the following sub-sections, we will discuss HetNet, user association, and load balancing in detail and discuss some open problems.

### 1.1. HetNet and User Association

To boost areal spectral efficiency, reuse of radio resources is indispensable. However, the deployment of more macro BSs, forming a dense homogeneous network, is an unattractive solution as the deployment costs, along with the operational and maintenance costs, are very high. An alternative to this homogeneous deployment is to have a heterogeneous one, where many low-powered small cells are deployed within the coverage area of a macro BS. A HetNet cellular network consists of randomly located BSs forming a multi-tier cellular network. Here, each tier differs in the average transmitting power, data rate, and deployment density. The HetNet provides big capacity, high performance, ease of operation, and fast deployment, but with the cost of additional revenue, interference, resource management, and backhaul management [13]. HetNet also has the potential to degrade load imbalance among different BSs due to disparate transmit powers and capabilities [14]. However, with HetNet environment, there are some critical challenges, such as *user association*, *power control*, and *resource allocation* that need to be addressed [15]. The traditional cell-selection strategy, max-SINR(signal-to-interference-plus-noise ratio) [16], used by user equipment (UEs) causes load imbalance in HetNet [17]. This is mainly because UEs in the coverage area of small BSs still receive the strongest signal from the macro BS, and also because the varying data-traffic pattern in the network is not being considered during the cell-selection procedure. To overcome this problem, UEs should be effectively pushed onto the small BSs, but currently, cellular standards support max-SINR, max-RSSI (received signal strength indicator) [16], and biased received power-based user association [18] schemes. In the first two schemes, the UE will be associated with the BS from which it receives maximum signal strength among all the BSs. From the UE’s perspective, this scheme should give a better data rate. In a biased received power-based user association scheme, UEs are offloaded to low-powered small cells using association bias [14]. The UE adds a bias value to the receiving power of the low-powered small cells; however, it is difficult to prescribe the optimal biased value. These schemes perform well in the traditional cellular network [14], where the macro BSs have uniform transmitting power and are deployed uniformly. However, the HetNet environment is completely different, as the heterogeneous BSs are not deployed uniformly.

### 1.2. Load Balancing Problem

The definition of “load” in a cellular network changes according to the environment. The authors in Refs. [14,17,19] consider the load of a BS as the number of UEs attached to it. If the maximum number of UEs supported by a BS varies and the average time frequency resource used by UEs is constant, then the traffic flow experienced by the BS is directly proportional to the attached number of UEs. This definition of load is simple to consider and easy to implement. If a BS has many UEs attached to it, then the total resources will be divided among the attached UEs. Hence, the per-user data rate gets reduced, which is not desirable from the UEs perspective. Authors in Ref. [20] consider the application-aware target rate and instantaneous physical rate to calculate the load of a BS. The UE’s satisfaction index is calculated by taking the inverse of the load. If a UE is associated with a highly loaded BS, then it will be less satisfied. Traditional user association schemes in a HetNet environment with small cells result in lower UE experience, as well as load imbalance in the network; hence, a novel user association scheme should be adopted. Thus, our objective is to design a novel user association and load-balancing model in the new HetNet environment.

In this paper, we propose low-complexity online distributed algorithms for cell selection in a HetNet environment, while considering two types of load definitions. The BSs and UEs are treated as bidders and objects, respectively, such as that in online combinatorial auctions. We present an analytical model to show the near-optimal performance of our proposed solutions.

The remainder of this paper is organized as follows. Section 2 presents some related works. In Section 3, the system model is presented and the problem is formulated. Distributed cell-selection algorithms are presented in Section 4. Performance analysis is given in Section 5. The performance evaluation, discussion, and conclusion are given in Section 6, Section 7 and Section 8, respectively.

## 2. Related Works

Recent surveys [14,21] show different schemes regarding user association for load-balancing in a HetNet environment. In Ref.  [14], the authors surveyed different schemes to solve the load-balancing problem, such as centralized optimization, the game theory, Markov decision process, and biasing schemes, and also mentioned some of the open challenges. They also discussed a few myths in detail, such as signal quality (which is the main driver of user experience) and spectrum crunch. UE will not always get the best experience from the strongest BS in its range, and the current focus should be on the development of infrastructure rather than spectrum shortage. The authors in Ref. [22] surveyed various user-association schemes and summarized the challenges into four parts, being complexity, energy efficiency, spectrum efficiency, and interference management. They also stated that the large number of UEs, together with the increasing number of BSs requires simple user-association algorithms, with minimal signaling and processing overhead. Authors in Ref. [23] proposed a load-balancing scheme based on a machine learning technique that uses both unsupervised learning and supervised learning, as well as the Markov Decision Process. Authors in Ref. [21] discussed the control mechanisms for user association in a cellular network which can be divided into three categories, namely centralized control, distributed control, and hybrid control. In centralized control, a single processing unit takes all the decisions for user association. Some centralized controls were presented in Refs. [24,25,26,27]. The authors in Ref. [24] formulated the resource allocation with a load-balancing problem as a mixed-integer nonlinear non-convex combinatorial optimization problem. Then, a nonlinear fractional programming scheme and dual decomposition scheme were applied to search the optimal solutions. A two-tier scenario with a centralized controller for load-balancing was considered in Ref. [25], where the load-balancing problem was formulated as a nonlinear integer problem, and a centralized entity was responsible for the load-balancing task. The authors in Ref. [26] proposed an ON-OFF transmission coordination model which can control the BS to either transmit at the maximum power or none. An optimization problem was formulated to maximize the throughput and balance the load in Ref. [27], where the user-association task was done centrally. Centralized controls have been proven to show good results in terms of optimal resource allocation, with a fast convergence. However, the required amount of signaling overhead grows exponentially, and is intractable with the increase in network size [17,21]. Hence, a scheme having decentralized implementation with low-signaling overhead is desirable [28]. Distributed controls [17,19,20,29,30,31,32,33,34,35,36,37,38,39,40,41,42,43] can be further divided into the static/offline and dynamic/online environment, and are guaranteed to be the simple and low-complexity alternative to centralized controls. In a distributed control, the UEs select the BSs based on the received signal strength and information received, such as load information, from BSs in range. BSs and UEs make autonomous user association decisions by themselves through mutual interaction. Both the BSs and the UEs execute the load-balancing algorithm on their respective ends. The BSs broadcast the load parameter periodically, and the concerned UEs take the decision of associating to a BS based on these load values and signal strengths. These schemes achieve higher performance in terms of network throughput and load balance.

### Offline and Online User Association

In the offline user association scheme, a static network environment is provided where the complete setup is assumed to be known previously. In general, a throughput optimization problem is formulated for the user association problem. Authors in Refs. [17,31,32,33,34,35,37,38,39,40,41,42,43] considered a static environment, where the channel condition does not change. A q-learning-based BS selection game was applied in Ref. [30]. In this, the UE tries to maximize a utility function by associating it with the best possible candidate for BS. The system converges to Nash equilibrium after several numbers of steps. By considering numerous sensors and mobile devices with a dense deployment and formulating the problem as a non-cooperative game mode, authors in Ref. [38] investigate the cell selection problem. They theoretically found the circular boundaries between the devices selecting the macrocell and those selecting the picocells. However, in the game-theory-based schemes, the players (BSs or UEs) cannot act rationally all the time due to the fact that different players (e.g., BSs) always have different optimization objectives [44]. Although the user association problem for a static/offline environment has been thoroughly investigated, less attention has been given to the online user association. The motivation behind the online user association problem is the practical nature of the problem. This is due to the fact that, in the presence of new UEs arriving over time, the problem is solved from scratch, where there is a high probability of UE re-association. This is not desirable from a UE’s perspective, where frequent re-association occurs. The online schemes, on the other hand, do not need the complete setup in prior. The association decision is taken in an online fashion—that is, each UE associates with a BS upon arrival to the network. There is less related work present for the online user association problem. Authors in Ref. [19] highlighted the problem in static environments, and proposed user-centric and cell-centric online algorithms. A game-theory-based BS selection strategy was investigated in Ref. [39]. The problem was formulated as a non-cooperative game, where the strategy converges to Nash-equilibrium in a small number of steps. These schemes are straightforward as the maximum of a utility is considered for the user association. In this scheme, UEs arrive to the system one after another and select the BS which either maximizes the utility of the BS or the UE. Authors in Ref. [45] study the user association problem by considering both energy and spectrum efficiency maximization of the 5G HetNet. Taking the quality of service into account, the authors develop a low-complexity algorithm to solve the formulated optimization problem in an approximate manner. The approximation algorithm can also be employed in an online fashion. In Table 1, we have compared various user association schemes, while considering fairness and the environment type.

The low complexity and dynamic nature of the distributed algorithm is well-suited for the cell selection problem. In this paper, we proposed two types of online distributed algorithms for load-balancing in a heterogeneous cellular network, such as *probabilistic* and *d-choices*-based user association schemes. *D-choices*-based distributed load-balancing algorithms have been successfully applied to various server selection and channel allocation problems [46], where a client chooses *d* number of servers uniformly at random and associates with that server, which has a minimum load. A solution to balls into non-uniform bins was investigated in Ref. [47], where a heterogeneous environment was considered and the balls are assigned to the bins. After the allocation, the maximum load (number of balls) was found to be $\frac{lnln(n)}{ln(d)}$, where *n* is the number of bins and *d* is the number of choices for a ball. A more realistic environment was considered in Ref. [48],where a ball arrives in a random location and wishes to associate with a bin, which is the same as the user association problem. The procedure achieved a balanced allocation after the execution of the scheme. In the heterogeneous RAN, a tier-wise, as well as BS wise load-balancing is desirable, and hence a multi-level load-balancing scheme is needed. To the best of our knowledge, a *d*-choices-based scheme has not been applied to the user association problem in a cellular network.

## 3. System Model and Problem Formulation

We consider a ***K-***tier HetNet, and denote the set of tiers as K = {1,2,...,K}, the set of all BSs as B = {1,2,...,B}, and the set of UEs as U = {1,2,...,U}. The cardinality of B and U give the total number of BSs and UEs in the system, respectively. Each small BS uses a wireless backhaul to connect with the macro BS, either directly or indirectly. We adopt a universal frequency reuse scheme among the different tiers. This model can easily be extended to a multi-RAT scenario. We assume that at a given point of time, a fraction of the total UEs, say U, will get access to the network. Without the loss of generality, the UEs are indexed as their arrival in the system—that is, where UE 1 arrives first and UE ***U*** arrives last. The maximum capacity, as well as maximum transmission power varies from one tier to another. Each BS has a positive integer capacity, also termed the size of a BS. We denote $\lambda_{j,k}$ and $P_{j,k}$ as the current load and the transmission power respectively of BS *j* at tier *k*, where *j* ∈ B and *k* ∈ K. To calculate the load of a BS, we consider two different parameters—the number of UEs and the SINR experienced from a BS. While considering the number of UEs as a load parameter, we consider the following assumptions. Each UE in the network consumes a portion of the time-frequency resource from the BS. Here, we consider an orthogonal frequency-division multiple access (OFDMA)-based medium access control (MAC) protocol with fair sub-carrier scheduling, as given in Ref. [27]; hence, the load of the BS could be the total number of UEs associated with it [17]. The average data rate from a BS also signifies the load, as the highly loaded BS’s average data rate will be lower, as compared with other BSs. We present $load_{1}$ and $load_{2}$ as the loads occurring due to the number of UEs and the average data rate of the BS, respectively. In a HetNet environment, BSs of different tiers can provide services to a varying number of UEs, and come with varying capacities. For example, a macro BS can offer services to a considerably large number of UEs, as compared to a pico BS. If we consider the same number of UEs attached to both macro BS and pico BS, then the load is not same for both the BSs; in contrast, the macro BS is lightly loaded as compared to that of pico BS. One important parameter, SINR, plays a key role in accessing the network. At first, a UE scans all the possible BSs it can access, and then it calculates the SINR, $\eta_{i,j,k}$, of these BSs:

(1) $$\begin{array}{c} \eta_{i,j,k}=\frac{P_{j,k}h_{i,j,k}}{\sum_{l\in B,l\neq j}P_{l,k}h_{i,l,k}+\sigma^{2}}\;, \end{array}$$

where $P_{j,k}$ is the transmission power of BS *j* at tier *k*, $h_{i,j,k}$ is the channel gain between UE *i*, and BS *j* at tier *k*. $\sigma^{2}$ denotes the noise power level, and $\sum_{l\in B,l\neq j}P_{l,k}h_{i,l,k}$ is the interference received from other BSs of the same tiers. The instantaneous data rate, $r_{i,j,k}$, associated with the SINR, $\eta_{i,j,k}$, is given by:

(2) $$\begin{array}{c} r_{i,j,k}=W\log_{2}\left(1+\eta_{i,j,k}\right) \end{array}$$

In a conventional network, the UE will associate with that BS whose SINR is the maximum, among all the BSs in range. In a HetNet environment this type of association scheme will lead to load imbalance among BSs. UEs attached to macro BS will not get the desired data rate, though the instantaneous data rate is quite high. This is because the total resources will be divided among all the associated UEs, and the per-user data will be significantly low. Let $N_{j,k}$ be the set of UEs attached to the BS *j* of tier *k*. If the BS schedules for equal resources for each UE in an OFDMA-based MAC protocol [17,19,27,39], then the actual UE data rate will be given by the following equation:

(3) $$\begin{array}{c} R_{i,j,k}=\frac{r_{i,j,k}}{|N_{j,k}|}, \end{array}$$

where $|N_{j,k}|$ is the total number of UEs attached to BS *j* at tier *k*. Note that if a scheme can evenly distribute UEs among the BSs, then the minimum data rate will also increase as the per-user resource sharing rate will be high.

We assume that equal time-sharing will be used to schedule the UE, in case a BS is associated with multiple UEs. Hence, the throughput of an UE *i* depends on the total number of UEs attached to and sharing the same BS [17,19,39]. While defining the $load_{1}$, we consider the current load of a BS to be the number of attached UEs. The $load_{1}$ of a BS is defined as follows:

(4) $$\begin{array}{c} \lambda_{j,k}^{load1}=\left|N_{j,k}\right|, \end{array}$$

Similarly, while considering the average data rate of a BS as $load_{2}$, we have the following expression,

(5) $$\begin{array}{cc} & \lambda_{j,k}^{load2}=\frac{\sum R_{i,j,k}}{|N_{j,k}|}. \end{array}$$

Note that both Equations (4) and (5) represent the load of a BS by considering different parameters. One important difference in $\lambda_{j,k}^{load1}$ and $\lambda_{j,k}^{load2}$ is that, the higher $\lambda_{j,k}^{load1}$ value signifies a higher load of a BS, while a higher $\lambda_{j,k}^{load2}$ value implies a lower load. The definitions of all the variables are given in Table 2.

Before the problem statement is formally defined, we highlight the trade-off problem between throughput and fairness. The authors in Ref. [49] presented a family of fairness functions by considering five axioms of fairness measures. Jain’s fairness [50], α-fairness, and entropy are special cases of this family. Furthermore, authors in Refs. [51,52] provide an analytical framework of a throughput-fairness trade-off, and show that an approach can be considered more or less fair compared to other ones, depending on the fairness definition. In this work, we maximize the throughput of the network by considering the log utility function. This ensures that allocation of more resources for a well-served UE has a low priority compared to the allocation of more resources to UEs with low data rates [17].

### Problem Formulation

Taking a utility function perspective, we assume that UE *i* obtains utility $U_{i}\left(R_{i,j,k}\right)$ when the receiving data rate is $R_{i,j,k}$, which is a function of the actual data rate $R_{i,j,k}$. Let $x_{i,j,k}$ be the association indicator, $x_{i,j,k}$ = 1 if user *i* is connected to the BS *j* of tier *k* and 0 otherwise. We formulate the user association problem for $load_{1}$, that maximizes the aggregate utility function (denoted as **Q1**):

(6) $$\begin{array}{cc} & \underset{x_{i,j,k}}{\mathrm{maximize}}\;\sum_{i\in U}\sum_{j\in B}\sum_{k\in K}x_{i,j,k}U_{i}\left(\frac{r_{i,j,k}}{\sum_{l\in U}x_{l,j,k}}\right) \end{array}$$

(7) $$\begin{array}{c} s.t.\;\sum_{j\in B}\sum_{k\in K}x_{i,j,k}=1,\;i\in U \end{array}$$

(8) $$\begin{array}{c} \;\eta_{i,j,k}\geq \tau ,\;i\in U,\;j\in B,\;k\in K \end{array}$$

(9) $$\begin{array}{cc} & \;x_{i,j,k}\in \left\{0,1\right\},\;i\in U,\;j\in B,\;k\in K, \end{array}$$

where the constraints ensure that a UE can only be associated with a single BS, and the SINR should be greater than some threshold τ. The SINR threshold τ is the minimum SINR level required to decode the received signal from the BS. For example, the SINR required to decode a Quadrature Phase Shift Keying (QPSK)-modulated signal is comparatively lower than that of a Quadrature amplitude modulation (QAM)-modulated signal. The association indicator, $x_{i,j,k}$, ensures that a UE can only connect to a single BS. This problem can be easily extended to multiple BS association. Since the above problem **Q1** is an integer program, it is generally difficult to find the optimal solution. The problem **Q1** can easily be converted to a convex optimization problem by simply making the utility function as a logarithmic utility and relaxing the constraint in Equation (9) as $x_{i,j,k}\geq 0.$ This converted convex optimization problem can be solved effectively. The above optimization problem, **Q1**, can be reconfigured, which maximizes the average utility function (denoted as **Q2**) as follows:

(10) $$\begin{array}{cc} & \underset{x_{i,j,k}}{\mathrm{maximize}}\;\sum_{i\in U}\sum_{j\in B}\sum_{k\in K}x_{i,j,k}\frac{U_{i}\left(\frac{r_{i,j,k}}{\sum_{l\in U}x_{l,j,k}}\right)}{|N_{j,k}|} \end{array}$$

The problem **Q2** maximizes the average UE throughput, which ensures fairness in the network. The problem **Q2** has same set of constraints as that of **Q1**.

## 4. Algorithm Description

**Figure 2:**
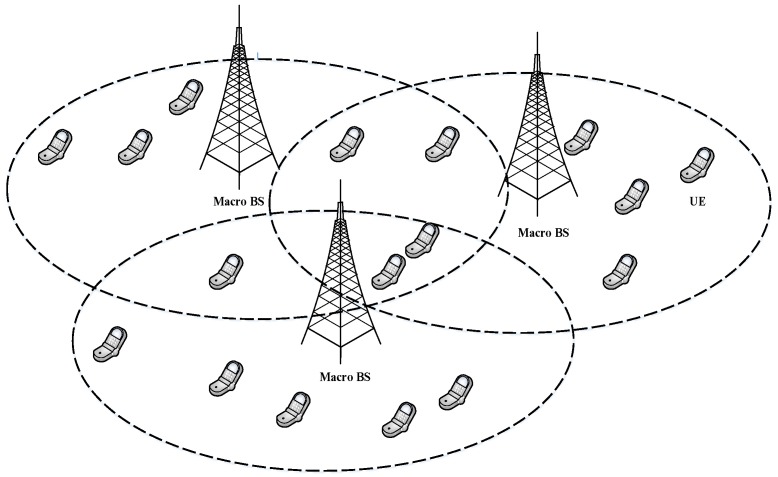
User equipments (UEs) placed in a homogeneous environment. Some UEs can access two or more macro base stations (BSs).

In the following, we consider a static environment and implement a centralized solution, and later compare with our proposed schemes to show the novelty. Figure 2 shows a static environment, where the UEs have no mobility and the channel condition remains constant. The centralized entity requires some primitives before making any decisions. Below are the numbers of functions with their meanings, which are essential to running the centralized solution.

1. $Neighborhood(BS_{j})$ = Set of BSs neighbors to BS *j*. 1≤j≤B
2. Can_Access(i) = Number of BS(s) that can be accessed by the *i*th UE. 1≤i≤U

The UE obtains the Can_Access(i) value by performing an initial BS search. The BSs whose SINR is greater than a threshold, τ, are considered. UEs are responsible for sending the Can_Access() value to their respective BSs, and the BSs, in turn send these parameters to the centralized entity. Algorithm 1 is then executed at the centralized entity, where it searches for the BS having optimum $\lambda_{j,k}$ value at each loop. By optimum $\lambda_{j,k}$, we mean the minimum for $\lambda_{j,k}^{load1}$ and maximum for $\lambda_{j,k}^{load2}$. Upon finding the BS, the centralized entity searches for neighboring BSs which are having optimum $\lambda_{j,k}$ values. Then, a UE selection strategy is applied to select a particular UE. UE whose Can_Access() value is 2 is given higher priority over the UE whose Can_Access() value is 3 and so on, because Algorithm 1 gives priority to UEs which have a lesser number of BSs in its range. As we consider two load values, $\lambda_{j,k}^{load1}$ and $\lambda_{j,k}^{load2}$, a proper decision must be taken for the BS selection at steps 2 and 4 of the algorithm. For $\lambda_{j,k}^{load1}$, min is considered at step 2 and max is considered at step 4, while for $\lambda_{j,k}^{load2}$ the opposite decisions are taken. This is because of the nature associated with $\lambda_{j,k}^{load1}$ and $\lambda_{j,k}^{load2}$, where higher value of the former gives a higher load, while the opposite holds true for the latter.

**Algorithm 1** Centralized solution
1:Assign UEs to the BSs which have Can_Access()=1;2:Find the BS with a opt $\left(\lambda_{j,k}\right)$ value,  L= opt $\left\{\lambda_{j,k}\right\}$, ∀j∈B,∀k∈K3:
$L_{nh}=neighbourhood\;\left(L\right)$
4:Find a BS which has a opt $\lambda_{j,k}$ value and is a neighbor to BS *L*,  S= $opt\;\{\lambda_{j,k}|j,k\in L_{nh}\},$ ∀j∈B,∀k∈K5:
**if** L∩S=ϕ **then**
6:  $L_{nh}=L_{nh}-\left\{S\right\}$7:  **if** $L_{nh}=\phi$ **then**8:    Do not consider this base station in future processing9:    Go to Row 210:  **end if**11:  Go to Row 412:
**else**
13:  $A=L_{nh}\cap S,c=2$14:  Select an UE *i* whose Can_Access(i)=c,i ∈ *A*15:  **if** Can_Access(i)=ϕ **then**16:    c=c+117:    Go to Row 1418:  **end if**19:  Assign the Selected UE to BS $L_{nh}$20:
**end if**
21:Update the $\lambda_{j,k}$, ∀j∈B,∀k∈K22:Goto Row 2


The above-mentioned scheme works well in a static, idealistic environment where there is no mobility and the channel quality remains intact. A slight change in the Can_Access() value triggers the centralized entity to re-run the algorithm from scratch, which is not suitable for a dynamic case. Hence, we develop distributed methods to handle the dynamic nature of the environment. Two schemes are developed for UEs with mobility using a distributed scheme: (a) the probability-based and (b) the *d*-choices-based selection, which try to balance the load over the whole network.

### 4.1. Probability Scheme

In this probability scheme, the UE selects a BS in a probabilistic manner upon arrival at the network. Actually, the UE considers two parameters, $\eta_{i,j,k}$ and $\lambda_{j,k}$, before associating with a BS *j* at tier *k*. Higher values of $\eta_{i,j,k}$ and optimal $\lambda_{j,k}$ yield in higher probability of associating with the BS *j*. The intuition behind this scheme is that higher $\eta_{i,j,k}$ from the BS gives a higher data rate, and optimal $\lambda_{j,k}$ of the BS leads to better down-link speed. The working principle of the probability scheme is easy to describe. The BSs periodically transmit their load parameter. When a new UE *i* arrives at the system, it checks for the BSs in range and calculates the $\eta_{i,j,k}$, and also receives the $\lambda_{j,k}$ parameter of these BSs. Let *b* be the set of BSs the UE can associate with. For each BS that satisfies the SINR constraint τ, the UE *i* calculates the following association parameter $\alpha_{i,j,k}$:

(11) $$\begin{array}{c} \alpha_{i,j,k}:=fun\left(\eta_{i,j,k},\lambda_{j,k}\right) \end{array}$$

The value of $fun(\eta_{i,j,k},\lambda_{j,k})$ for $\lambda_{j,k}^{load1}$ and $\lambda_{j,k}^{load2}$ is given by $U\left(\frac{r_{i,j,k}}{|N_{j,k}+1|}\right)$, and $U\left(r_{i,j,k}*\frac{\sum R_{i,j,k}}{|N_{j,k}|+1}\right)$, respectively. Note that U(.) represents the UE’s own utility. After calculating the $\alpha_{i,j,k}$ for each BS in its range, the UE *i* then calculates the probability of association. The probability of association with a BS is given by:

(12) $$\begin{array}{c} \delta_{i,j,k}=\frac{fun(\eta_{i,j,k},\lambda_{j,k})}{\sum_{j\in b}\sum_{k\in K}fun\left(\eta_{i,j,k},\lambda_{j,k}\right)},i\in U,j\in B,k\in k. \end{array}$$

The UE now has information regarding the probability of association with each BS. Equipped with this information, the UE then selects one BS in a probabilistic way. Note that the BS having higher $\alpha_{i,j,k}$ will have a higher probability to be selected. Algorithm 2 depicts the above scheme.

**Algorithm 2** Probability scheme
1:Initialize $\delta_{i,j,k}$ := 0, *i* ∈ U, *j* ∈ B, *k* ∈ K2:**for** each UE *i* in the network **do**3:  $B_{i}$ := { set of BSs ∈ B | $\eta_{i,j,k}\geq \tau$ }4:  Calculate $\alpha_{i,j,k}:=fun\left(\eta_{i,j,k},\lambda_{j,k}\right)$5:  Associate UE *i* with BS *j* of tier *k* with probability $\delta_{i,j,k}$6:
**end for**



Each UE executes the Algorithm 2 at their respective end. In Step 1, all the UEs in the network initialize their association probability to 0. Cell selection begins at Step 2 and in Step 3, the UE collects the BS information, which satisfies the SINR threshold. These are the potential BSs the UE can attach to. As the BS broadcasts their load information periodically, the UE is able to calculate $\alpha_{i,j,k}$ for each BS in $B_{i}$ (Step 4). The expression $fun(\eta_{i,j,k},\lambda_{j,k})$, given at Equation (11), is different for $\lambda_{j,k}^{load1}$ and $\lambda_{j,k}^{load2}$. The UE associates with a BS in $B_{i}$ with a probability $\delta_{i,j,k}$ according to Step 5.

### 4.2. Hierarchical *d*-Choices Scheme

The user association problem can be thought of as the “balls and bins” problem, shown in Ref. [48]. We can consider UEs to be balls and BSs to be bins. Each UE has a certain BS to associate with, and each BS has a specific capacity—for example, macro BS has more capacity than that of pico BS. Like in the traditional “balls and bins” experiment, each ball can choose *d* number of bins to associate with, and the expected maximum load is given by Equation (13), where *n* is the number of balls in the system. Here, O(1) represents a constant. No matter how large the size of *n* be, the maximum load is bounded by $\frac{\ln\ln n}{\ln2}$.

(13) $$\begin{array}{c} E\left[\mathrm{maximum}\;\mathrm{load}\right]=\frac{\ln\ln n}{\ln2}+O\left(1\right). \end{array}$$

The same principle can be applied to the association scheme in a cellular network. Each user can choose *d* number of BSs and select a BS that has the lightest load among the chosen BSs. This above method is well-suited for homogeneous cases, where each BS has the same capacity and same probability of association, but in the case of HetNet, the capacity varies from one tier to another. Hence, load-balancing in this HetNet should be carefully considered. Authors in Ref. [47] proposed a “balls into non-uniform bins” method. Accordingly, we first reduced our BS selection problem to the “balls into non-uniform” problem, and then applied the algorithm prescribed therein. Each UE will associate with a BS so that the final load will be bounded by some factor.

The *d-choices* scheme has been successfully applied to many load-balancing applications. The main strategy is that when a UE arrives to the system, it chooses *d* servers in the system uniformly at random. Then, the UE checks the load of each of the *d* servers, and associates with the server with the least load. We tried to apply a similar scheme to this cellular network. Our scheme consists of two levels: (1) load-balancing in different tiers, and (2) load-balancing in each BS in a particular tier. The network traffic for different RATs must flow through the corresponding service gateway, so care must also be taken to balance the load in the service gateway level. We assumed that all BSs broadcasted their load information periodically. The macro BS, having the highest coverage area, has a special task, in that it accumulates all the load parameters of BSs of different tiers, adds them up tier-wise, and then broadcasts the load value. The broadcast signal by macro BS will have the load value of all tiers in the network.

**Figure 3:**
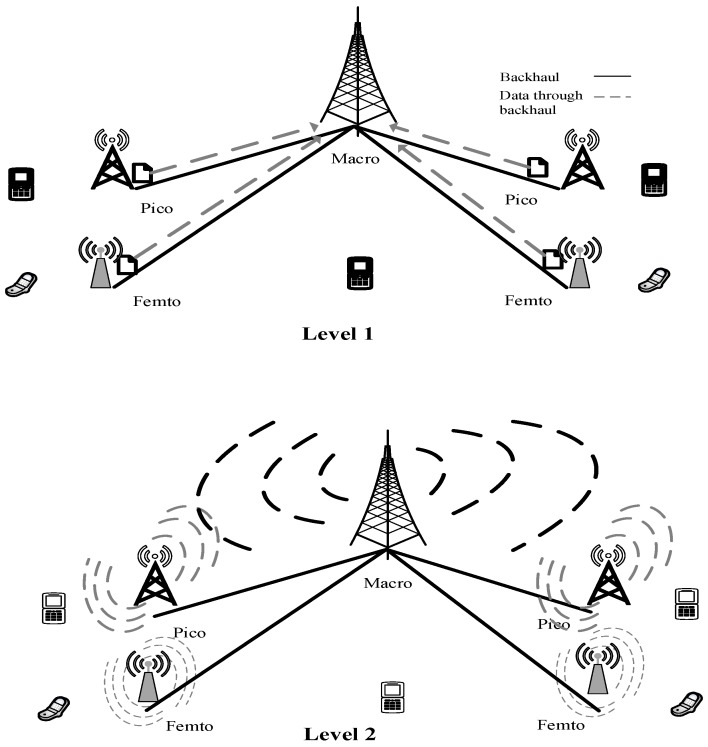
Two-level load transmission. In Level One, all the small-cell BSs, pico and femto, send their respective load value to macro BS. In Level Two, all the base stations (BSs) broadcast their load values.

The UE performs the load-balancing task in two levels. Figure 3 shows the procedure of load transmission in two levels. After receiving the load information from macro BS, it selects *d* tiers uniformly at random, and then chooses a tier from the *d* number of tiers which has a minimum load. The next step is to choose a BS from the already chosen tier. The UE repeats the above procedure for selecting a BS from the chosen tier. The UE chooses *d* number of BSs uniformly at random, and selects a BS from the *d* number of BSs which has a minimum load. This procedure ensures load balancing at two levels: (1) tier-level load-balancing, and (2) BS-level load-balancing. Note that tier-level load-balancing is required to shift the load from a heavily loaded tier to a lightly loaded tier. Algorithm 3, *d-choices*, depicts the above distributed *d*-choices scheme by considering $\lambda_{j,k}$.    

**Algorithm 3** Two-level *d*-choices scheme.
1:$\lambda_{i}$ := {set consisting loads of tiers for *i*th UE | $\eta_{i,j,k}\geq \tau$ }2:**for all** UEs, *i* **do**3:  Independently choose a set $K_{i}^{d}$ of d1 tiers at random from $\lambda_{i}$4:  $\lambda_{i,k}$ := {loads of tiers belong to $K_{i}^{d}$ }, without loss of generality $\lambda_{i,k}$ := { $\lambda_{i,1},\lambda_{i,2},...,\lambda_{i,d1}$ }5:  find $K_{\psi }$ := opt ( $\lambda_{i,1},\lambda_{i,2},...,\lambda_{i,d1}$ )6:  $B_{i}$ := { set of BSs ∈ $k_{\psi }$ | $\eta_{i,j,k}\geq \tau$ }7:  Independently choose a set $B_{i}^{d}$ of d2 BSs at random from $B_{i}$8:  $\lambda_{j,k}$ := {loads of BSs belong to $B_{j}^{d}$ }, without loss of generality $\lambda_{j,k}$ := { $\lambda_{j,1},\lambda_{j,2},...,\lambda_{j,d2}$ }9:  find $B_{\psi }$ := opt ( $\lambda_{j,1},\lambda_{j,2},...,\lambda_{j,d2}$ )10:   Associate UE *i* with $B_{\psi }$ BS11:
**end for**
12:$K_{\psi }^{load1}$ = $min\{\lambda_{i,1}^{load1},\lambda_{i,2}^{load1},...,\lambda_{i,d1}^{load1}\}$13:$K_{\psi }^{load2}$ = $max\{\lambda_{i,1}^{load1},\lambda_{i,2}^{load1},...,\lambda_{i,d1}^{load2}\}$14:$B_{\psi }^{load1}$ = $min\{\lambda_{j,1}^{load1},\lambda_{j,2}^{load1},...,\lambda_{j,d2}^{load1}\}$15:$B_{\psi }^{load2}$ = $max\{\lambda_{j,1}^{load1},\lambda_{j,2}^{load1},...,\lambda_{j,d2}^{load2}\}$


Algorithm 3 executes the *d*-choices schemes in two levels. The UE begins its user association procedure by accumulating the BSs which satisfy the $\eta_{i,j,k}\geq \tau$ in a tier-wise manner (Step 1). Subsequently, in Steps 3–5 the UE selects a tier from the $K_{j}$ set using the *d*-choices scheme. The same procedure is applied to choose a BS from the chosen tier in Steps 6–9. Then in Step 10, the UE associates with the chosen BS. As the two load parameters, $\lambda_{i,k}^{load1}$ and $\lambda_{i,k}^{load2}$, have different properties, the opt function in Steps 5 and 9 returns different values, as mentioned in Steps 12–15. In the next subsection, we discuss the effect of different values for d1 and d2.

#### Effects of Different *d*-Choices and Different User Association Methods

To evaluate the effect of different *d*-choices, we consider a hypothetical scenario where there are four tiers and each tier has 10 servers. 1000 UEs arrive at the system one by one to attach with a server. First, the UE randomly picks d1 tiers, and chooses the tier with the smallest load (total number of UEs). After the selection of an appropriate tier, the UE then randomly picks d2 servers from a selected tier and picks the server with the minimum load. We chose Jain’s fairness index for the fairness evaluation. The different *d*-choices, average Jain’s fairness index, and the total probes made by a UE are given in Table 3. The Jain’s fairness index with only four probes is about 0.9985, and this fairness does not increase significantly with an increase in the number of probes. The author in Ref. [53] showed that the power consumption of a UE increases with an increase in the number of probes.

Apart from selecting the tier with a minimum load, a new scheme could be considered for the evaluation. If we select the most unbalanced tier from the *d* chosen tiers and allocate the UE to the minimum loaded BS, then the scenario will be totally different. This strategy may not ensure a load-balancing scenario. Think of a situation where all the servers of a tier have an equal number of UEs, but the total UE number is less than another unbalanced tier. The balanced tier will never be selected, even though the total number of UEs are less than other tiers. The final outcome will be an unbalanced allocation; hence, this method is not desirable.

## 5. Performance Analysis

In this section, we give an analytic model of the performance for the online algorithms by considering $load_{1}$. The analysis for the $load_{2}$ follows the same line. We first prove the fact that the BS utility function is submodular and monotone. Then, we show that the UE utility function exhibits the same outcome as BS utility. As a concrete example, we analyze the logarithmic UE utility $U_{i}\left(.\right)$ = log(.), ∀i∈U, which is commonly used in wireless networks to provide proportional fairness among UEs [17]. The utility of the BS is defined as the sum utility of its associated UEs. Under the logarithmic utility, the BS utility function becomes:

(14) $$\begin{array}{c} V_{j,k}\left(N_{j,k}\right)=\sum_{i\in N_{j,k}}\log\left(\frac{\eta_{i,j,k}}{|N_{j,k}|}\right),j\in B,k\in K,N_{j,k}\subset \Omega_{j,k} \end{array}$$

**Definition** **1.** *The UE utility function $U_{i}\left(\cdot \right)$ is submodular and monotone.*

**Lemma** **1.** *$V_{j,k}\left(N_{j,k}\right)=\sum_{i\in N_{j,k}}\log\left(\frac{\eta_{i,j,k}}{|N_{j,k}|}\right)$, $k\in K,j\in B_{k},N_{j,k}\subset \Omega_{j,k}$ is submodular and monotone.*

**Proof.**  Our proof is, in essence, similar to the proof given in Ref. [19]. Here, we present it for completeness. For submodularity, we have the following definition:

For every N,O ⊆ Ω with N⊆O and every i∈Ω we have that V(N∪{i})-V(N) ≥ V(O∪{i})-V(O). Let us first consider the case N≠∅. We have

(15) $$\begin{array}{c} V_{j,k}\left(i|N\right)=V_{j,k}\left(N\cup \left\{i\right\}\right)-V_{j,k}\left(N\right).\\ =\sum_{l\in N\cup \{i\}}\log\left(\frac{\eta_{l,j,k}}{|N|+1}\right)\;-\sum_{l\in N}\log\left(\frac{\eta_{l,j,k}}{\left|N\right|}\right)\\ =\log\left(\eta_{i,j,k}\right)+|N|\log|N|-(|N|+1)\log(|N|+1). \end{array}$$

For $V_{j,k}\left(i|N\right)$ ≥ $V_{j,k}\left(i|O\right)$, is equivalent to |N|log|N|-(|N|+1)log(|N|+1) ≥ |O|log|O|-(|O|+1)log(|O|+1). To show the marginal gain, $V_{j,k}\left(.\right)$ is a decreasing function, and we need to show that nlogn-(n+1)log(n+1) is a decreasing function for n>0.

(16) $$\begin{array}{c} f(n)=n\log n-(n+1)\log(n+1)\\ f^{{}^{\prime }}\left(n\right)=\log\left(n\right)-\log\left(n+1\right) \end{array}$$

The function $f^{{}^{\prime }}\left(n\right)$ clearly is a decreasing function ∀n>0. For the other case *N* = ∅, we need to show that 0≥ |O|log|O|-(|O|+1)log(|O|+1). The proof is straightforward, as |O+1|≥|O| and log(|O+1|)≥log(|O|), ∀|O|>0. Thus, the expression $V_{j,k}\left(N\right)\geq V_{j,k}\left(O\right)$ holds true for all cases. We conclude that $V_{j,k}\left(.\right)$ is submodular. From (16), it is clear that $f^{{}^{\prime }}\left(n\right)$ is decreasing ∀n>0. Hence, $V_{j,k}\left(.\right)$ is monotonically decreasing. We conclude that $V_{j,k}\left(.\right)$ is *submodular and monotone.*

Now, we prove the fact that the UE utility function, $U_{i}\left(.\right)$, is similar to the marginal gain of the BS utility function—that is, $V_{j,k}\left(i|N\right)$. The UE *i*’s utility for BS *j* belongs to tier *k* is given as $\log\left(\frac{\eta_{i,j,k}}{\left|N+1\right|}\right)$, which is equal to

(17) $$U_{i}\left(.\right)=\log\left(\eta_{i,j,k}\right)-\log(|N+1|)$$

Now, we establish the similarity between Equations (15) and (17). Equation (15) can be re-written as:

$$V_{j,k}\left(i|N\right)=\log\left(\eta_{i,j,k}\right)-((|N|+1)\log(|N|+1)-|N|\log|N|)$$

**Figure 4:**
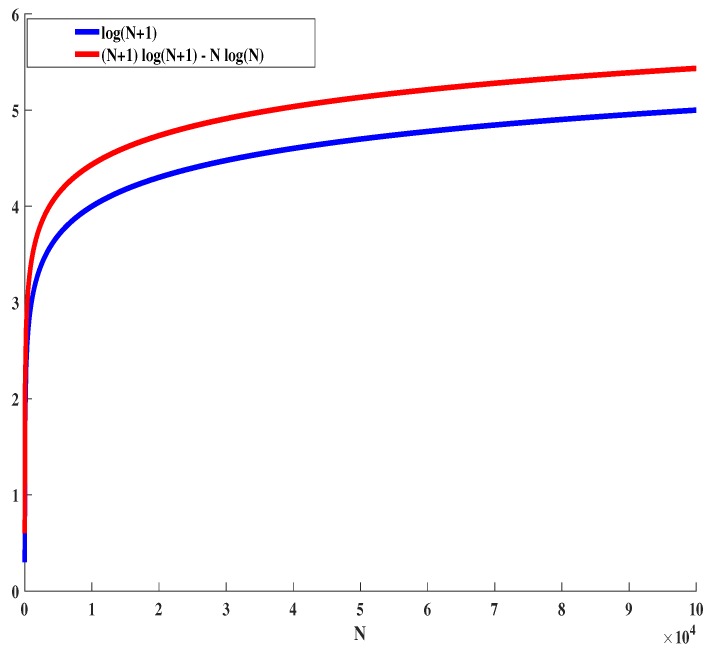
Similarity between UE’s utility and BS’s utility.

The expression log(|N+1|) from (17) and (|N|+1)log(|N|+1)-|N|log|N| from Equation (15) exhibits a similar trend. This can be verified from Figure 4. By following the same line after Equation (15), we conclude that the UE utility function, $U_{i}\left(.\right)$, is submodular and monotone. ☐

**Theorem** **1.** *Under the submodularity and monotonicity of $U_{i}\left(.\right)$, we have $E[ALG_{2}\left(Q_{1}\right)]$ ≥ $\frac{1}{2-b^{-1}}$$OPT(Q_{1})$, where $b=max_{i\in U}\left|B_{i}\right|$.*

**Proof.**  The submodularity and monotonocity of $U_{j}\left(.\right)$ are shown in the Lemma 1. By following the analysis of Theorem 1 in Ref. [19], we can conclude that $E[ALG_{2}\left(Q_{1}\right)]$ ≥ $\frac{1}{2-b^{-1}}$ $OPT(Q_{1})$. ☐

### Time Complexity Analysis

In this section, we compute the time complexity of the different proposed algorithms. The time complexity of Algorithm 2 depends on the number of UEs and BSs. Each UE calculates the $\eta_{i,j,k}$ and receives the $\lambda_{i,k}$ information from the candidate BSs; afterwards, it goes for the user association procedure. Calculation of $\eta_{i,j,k}$ and the probability of association for each BS depends on the factor that calculates how many BSs satisfy the SINR threshold. The number of BSs which satisfy the SINR threshold varies from UE to UE. We denote the average number of BSs accessed by these UEs to be *C*, where the time complexity for Algorithm 2 will then be of O(C), as the method is distributed in nature. Algorithm 3, on the other hand, works differently. Each UE selects a tier, *k*, in the first level and a BS, *j*, from that tier in the second level. Both selections of the tier and BS are performed on the number of choices d1 and d2, respectively. In Level 1, for the selection of a tier, the algorithm tries to find a tier that has a minimum load out of the d1 chosen tiers. This results in O(d1) for a single UE. In the second level of the algorithm, the UE finds a BS from the selected tier. The UE has a number of choices, but only d2 numbers of BSs are eligible for the access procedure. Therefore, the time complexity for Algorithm 3 depends on both levels 1 and 2. The time complexity of this distributed scheme is thus the sum of the two choices in two different levels. Hence, the time complexity is O(d1+d2). Table 4 shows the comparison of different algorithms by considering association rule, time complexity and scheme employed.

## 6. Performance Evaluation

**Figure 5:**
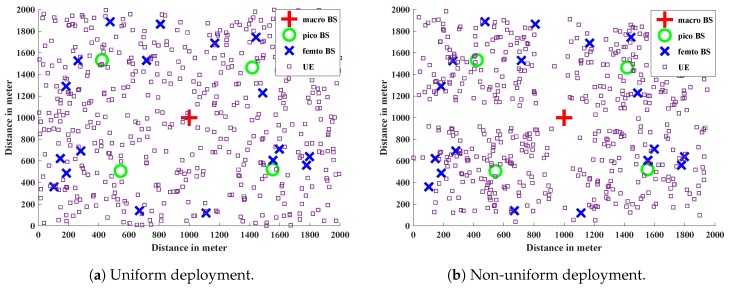
UE and BS deployment in the network.

Here, we consider a three-tier HetNet environment consisting of macro, pico, and femto BSs. We develop the HetNet environment and run the simulation on the MATLAB platform. The BSs are uniformly and independently distributed in a 2000 × 2000 m${}^{2}$ space. The macro BS is placed at the central location of the space, and 4 pico and 20 femto BSs are deployed in the whole region. We also consider a uniform and non-uniform deployment of the 500 mobile UEs in the network scenario. For non-uniform deployment, we place the UEs around Femto and Pico BSs. Figure 5a,b depicts the UE and BS deployment for uniform and non-uniform UE deployment respectively. These UEs arrive to the system online—that is, there is one UE arrival per unit time. The spectrum bands of different tiers are orthogonal to each other, and hence do not interfere with each other, whereas BSs which belong to the same tier do interfere. The transmission powers of macro, pico, and femto BSs are 46 dBm, 35 dBm, and 24 dBm, respectively. The path-loss model for macro BS is taken as 128 + 37.1*log${}_{10}$(*D*), where *D* is in kilometers. The path-loss model for pico and femto BS is taken as 37 + 30*log${}_{10}$(*D*), where *D* is in meters. We assume log-normal shadowing with a standard deviation $\sigma_{s}$ = 8 dB and a bandwidth of 10 MHz. The thermal noise power is $\sigma^{2}$ = −104 dBm. For the different *d* values in Algorithm 3, we restrict the choices to only two, as the probability for higher *d* decreases in a real environment [54]. We perform the simulation 100 times and averaged out the results. The following are the performance matrices used for the evaluation of our results. 

(a) Cumulative distribution function (CDF) of spectrum efficiency

(b) Normalized minimum user rate [19] 

The minimum user rate is normalized with the optimum value. The optimum value is the maximum among the minimum user rate on each scheme. Let *R* be the minimum data rate experienced by the UE(s) in the network. The value *R* will vary from one scheme to other. Let there be *N* schemes, so the normalized user rate, ${\tilde{\beta }}_{min}$, for each scheme, n∈N, is given by ${\tilde{\beta }}_{min}$ = $\frac{R_{n}}{max\{R_{1},R_{2},...,R_{N}\}}$. 

(c) Normalized sum user rate [19] 

The sum user rate of the network is normalized with the optimum value. The optimum value is the maximum among the sum user rate on each scheme. Let *S* be the sum user rate of the network. The value *S* will vary from one scheme to other. The normalized sum user rate, ${\tilde{\beta }}_{sum}$, for each scheme, n∈N, is given by ${\tilde{\beta }}_{sum}$ = $\frac{S_{n}}{max\{S_{1},S_{2},...,S_{N}\}}$. 

(d) Jain’s fairness [50] 

### 6.1. Loads among Different BSs

**Figure 6:**
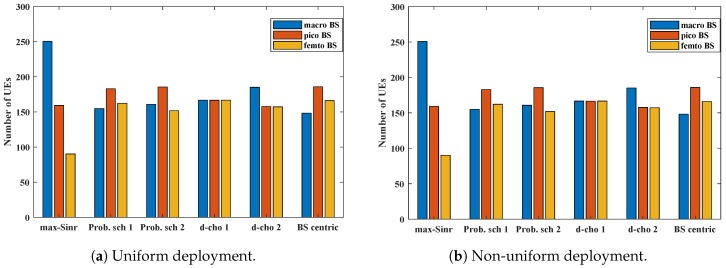
UE share for different association schemes in different deployment scenarios.

Along with the traditional *max-SINR*, we also consider the BS-centric algorithm (*BS-centric*), proposed in Ref. [19], as our benchmark scheme. The UE share in uniform and non-uniform deployment of UEs is shown in Figure 6a,b, respectively. In the uniform UE deployment scenario, as expected, the traditional *max-SINR* shows an uneven UE share. However, we can see that a considerable amount of UEs are being offloaded to Pico and Femto BSs in the case of *Prob. scheme 1*, *Prob. scheme 2*, and *BS-centric*. UEs associated with macro BS are being offloaded to both Pico and Femto BSs, which verifies the effectiveness of our proposed *probability scheme*s. The *d-choices* schemes, on the other hand, focus on tier-wise balanced UE allocation. Figure 6a clearly shows the balanced allocation of UEs for *d-choices 1* and *d-choices 2* schemes. A similar trend can be seen in non-uniform deployment. More UEs are offloaded to pico and femto BSs.

### 6.2. CDF of Spectrum Efficiency

**Figure 7:**
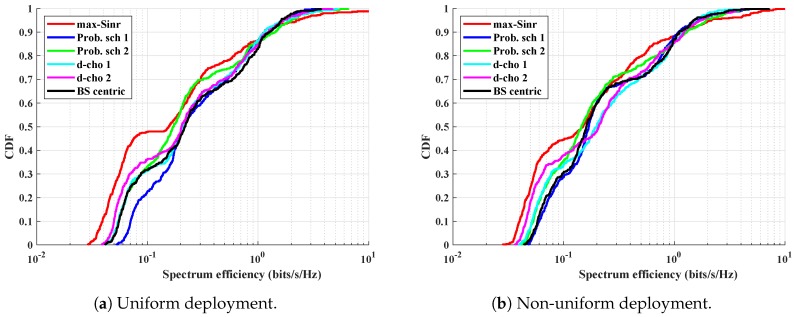
The cumulative distribution function (CDF) of spectrum efficiency for different association schemes in different deployment scenarios.

One way to give a better experience to the UEs experiencing low spectrum efficiency is to take the resource from strong UEs and assign it to weak UEs. This task of taking the resource from strong UEs is done through load-balancing. Figure 7a, for uniform UE deployment, shows the CDF of spectrum efficiency in HetNet for different association schemes. Approximately 85% of the UEs, for all the schemes, have a spectrum efficiency lower than 1 bit/s/Hz. This ensures that very few UEs close to the BS have a higher spectrum efficiency. *Prob. scheme 1*, *Prob. scheme 2*, *d-choices 1*, *d-choices 2*, and *BS-centric* schemes perform quite well at spectrum efficiency as compared to the traditional *max-SINR* scheme. When considering the *BS-centric* one, our proposed schemes give better service to the majority of UEs—approximately 65%—in terms of spectrum efficiency. In all the schemes (except the *max-SINR*) a majority of the UEs have a higher spectrum efficiency. Approximately 80% of UEs in all the schemes have higher spectrum efficiency, as compared to *max-SINR*. This is because the macro BS is highly loaded, and the total resource is divided among the UEs. This, in turn, lowers the spectrum efficiency of the associated UEs. For non-uniform UE deployment, although many UEs are placed around the lower-powered BSs, the outcome is similar to that of uniform deployment. All of our proposed schemes performed better in the mid-segment of the CDF graph, where the spectrum efficiency of the UEs are higher than that of *max-SINR*. Our proposed scheme, *Prob. scheme 1*, continuously performs better as compared to *BS-centric* for non-uniform deployment, as shown in Figure 7b.

### 6.3. Normalized Rate

**Figure 8:**
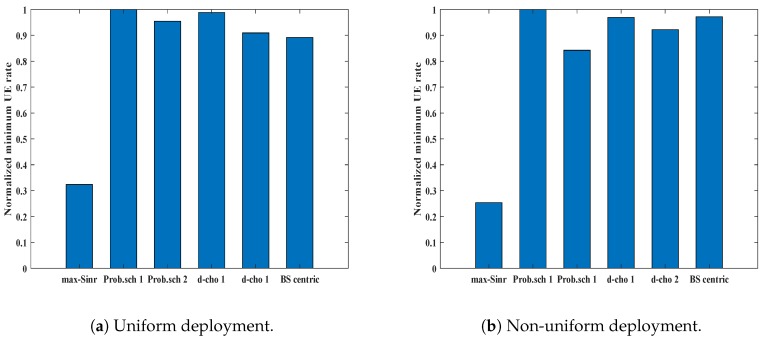
Normalized minimum rate for different association schemes in different deployment scenarios.

**Figure 9:**
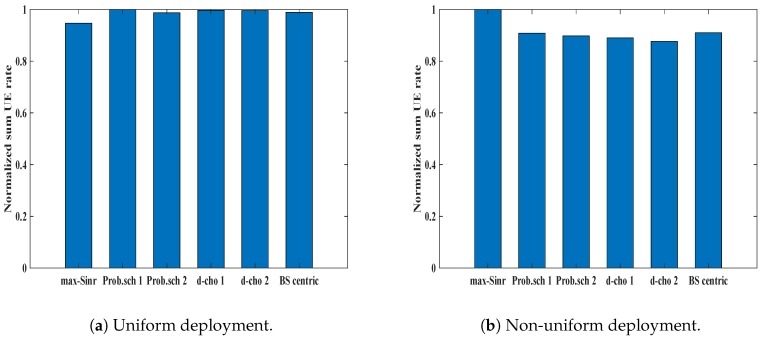
Normalized sum rate for different association schemes in different deployment scenarios.

Another performance metric for evaluation of the network performance is considering the normalized data rate of the UEs. The normalized minimum user rate, ${\tilde{\beta }}_{min}$ for different schemes in the uniform UE deployment scenario is shown in Figure 8a. ${\tilde{\beta }}_{min}$ for *max-SINR* is about 0.30, while *Prob. scheme 1* and *Prob. scheme 2* show improved performance. *Prob. scheme 1* shows a 70% improvement, while a similar trend can be seen for *Prob. schemes 2*. *d-choices 1* and *d-choices 2* show good results, and the minimum UE rate is higher, as compared to *max-SINR*. This gain in minimum user rate ensures that our scheme performs well for the cell edge UE, which mainly suffers with low data rate. All our proposed schemes perform better than *BS-centric* and *max-SINR*, while *Prob. scheme 1* achieves the highest minimum user rate. A similar trend can be seen in non-uniform deployment, as shown in Figure 8b. *Prob. scheme 1* continues to perform well in non-uniform deployment. The *max-SINR* scheme, on the other hand, continues to perform poorly. The metric “normalized sum rate”, denoted as ${\tilde{\beta }}_{sum}$, tells about the overall throughput of the network. In the case of traditional *max-SINR* schemes, the UEs near to the BSs receive higher signal strength. As can be seen in Figure 9a, our proposed methods for uniform deployment perform better than the *max-SINR*. On the other hand, for the non-uniform deployment, as shown in Figure 9b, the throughput of *max-SINR* performs slightly better. However, the *max-SINR* can achieve a higher sum user rate while ignoring the user fairness.

### 6.4. Jain’s Fairness

**Figure 10:**
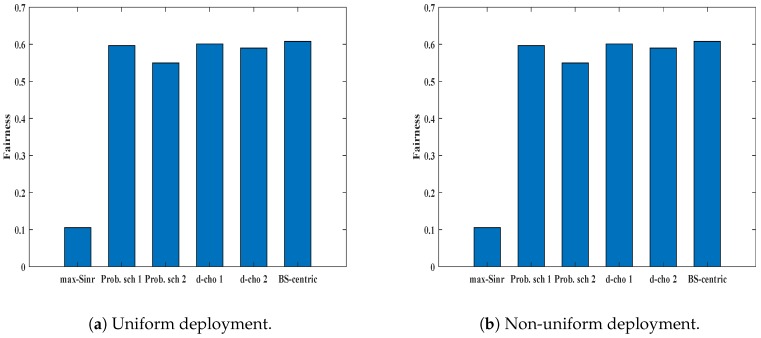
Jain’s fairness index of UE rate for different association schemes in different deployment scenarios.

Jain’s fairness index of UEs’ received data rate in a network shows the overall fairness. The fairness index becomes 1 when all UEs receive the same rate. Figure 10a,b shows the Jain’s fairness index of UEs’ received data rate for uniform and non-uniform deployment respectively. As expected, the Jain’s fairness index for *max-SINR* in uniform deployment is 0.1, which is very low. Unlike *max-SINR*, our proposed schemes perform better. The fairness index nearly equals to 0.6 for all the proposed schemes. The same outcome can be observed in the non-uniform UE deployment scenario.

**Figure 11:**
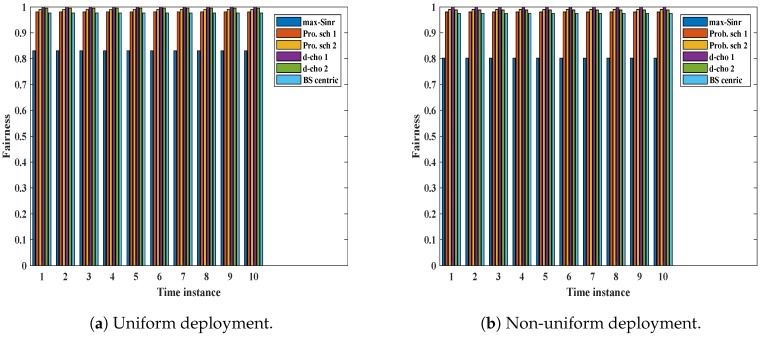
Jain’s fairness for different association schemes in different deployment scenarios.

User mobility is an unavoidable condition in a network. A network needs to perform well in a mobility environment. We considered Jain’s fairness index for the UE share in different schemes to show our performance on the mobility. A network is said to be balanced in a mobility environment if the fairness index is high in different time instances. We monitored the network for ten time instances and noted the fairness. We adopted a random walk for the UEs. Figure 11a shows the Jain’s fairness index in a mobility environment for uniform UE deployment. As soon as the UE changes its location and finds a better BS (higher SINR), it changes its serving BS. This type of association sometimes gets worse if all the UE moves closer to the macro BS. There is stability in *d-choices load1* and *d-choices load2* schemes. Each time the UE encounters a different load environment, it tries to balance the load of the network. For non-uniform UE deployment, the UEs are restricted to moving around the BS. Figure 11b shows the Jain’s fairness for non-uniform deployment. Both *d-choices* schemes continue to show good results, and there is slight poor performance by the *Prob. scheme*s, but it is better than *max-SINR*.

**Figure 12:**
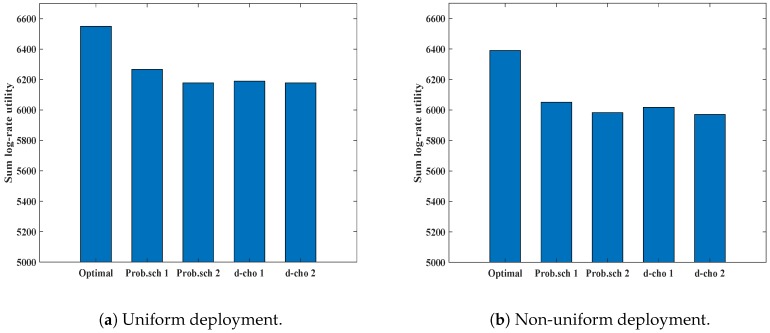
Performance of different association schemes in different deployment scenarios against optimal values.

In Figure 12a,b, we show a comparison of the performance of our proposed schemes with the optimal value. For the comparison, we consider the sum log-utility parameter. It is evident that the sum utility of our proposed schemes show near-optimal performance for both uniform and non-uniform deployment, which ensures the effectiveness of our proposed schemes.

## 7. Discussion

The spatial distribution of network elements, such as BSs and UEs, plays an important role for the calculation of different network parameters. From an analytical and tractability point of view, many researchers have considered the Poisson point process (PPP), a clustered point process for BS and UE deployment. The authors in Ref. [55] assumed a Poisson clustered process for the node location in a wireless ad hoc network. This kind of deployment is well-suited for high-demand areas where pico and femto BSs are clustered. The Ginibre point process (GPP) belongs to the class of determinantal point processes. It is part-way between the lattice and PPP. Hence, GPP exhibits a real-world scenario [56]. The studies in Refs. [56,57] present a network model based on GPP. Authors in Ref. [57] provided analytical proof to show that the GPP model allows higher accuracy compared to PPP. A similar kind of study is considered in Ref. [58], where the authors deployed the nodes using the Gauss-Poisson process. This distribution of the nodes illustrates the spatial distribution with attraction.

## 8. Conclusions

In this paper, we considered a three-tier HetNet environment, proposing user association schemes and dynamic load-balancing through *Prob.* and *d-choices* schemes. In the K-tier HetNet, the user association, conventionally connected to the BS with maximum SINR, results in less association with pico and femtocells. This ultimately leads to load imbalance in the whole network. Unlike the *max-SINR*, in the *Prob.* scheme, UE selects the BS in a probabilistic manner upon arrival into the network by considering both SINR and the load of a BS. This technique eventually offloads the UEs toward small cells. Inspired by the “balls and bins” load-balancing problem and the power of two choices, we proposed the *d-choices* association scheme. The UE can choose *d* number of BSs and selects the BS which has the lightest load among the chosen BSs. *Prob. scheme 2* showed better results in minimum user rate, while *d-choices 2* showed good results for tier-wise load-balancing in both uniform and non-uniform kinds of UE deployment. Load-balancing naturally provides the possible inclusion of more numbers of UEs in the network, typically giving better fairness and improving UE experience. These distributed schemes show an impressive result in dynamic environments by reducing the signal overhead between UE and BS, increasing the throughput per UE and network load balance. Future work includes the optimization of small cells in k-tier HetNet, while considering both energy and spectral efficiency with high user mobility. 

## Figures and Tables

**Table 1:** Comparison of user association schemes.

Ref.	Control	Environment	HetNet	Fairness
[17]	Distributed	Static	Yes	High
[19]	Distributed	Dynamic	Yes	High
[20]	Distributed	Dynamic	Yes	Moderate
[24]	Centralized	Static	Yes	-
[25]	Centralized	Static	Yes	High
[29]	Distributed	Dynamic	Yes	High
[26]	Centralized	Static	Yes	High
[27]	Centralized	Static	Yes	-
[30]	Distributed	Dynamic	Yes	-
[31]	Distributed	Static	No	-
[32]	Distributed	Static	No	-
[33]	Distributed	Static	Yes	High
[34]	Distributed	Static	No	-
[35]	Distributed	Static	Yes	-
[36]	Distributed	Dynamic	Yes	-
[37]	Distributed	Static	Yes	High
[39]	Distributed	Dynamic	Yes	-
[40]	Distributed	Static	Yes	-
[41]	Distributed	Static	Yes	High
[42]	Distributed	Static	Yes	-
[43]	Distributed	Static	Yes	-
[45]	Centralized	Dynamic	Yes	High

**Table 2:** Brief description of acronyms.

Notation	Description
K	Set of tiers
B	Set of BSs
U	Set of UEs
B	Number of BSs in the network
U	Number of UEs in the network
$\lambda_{j,k}^{load1}$	Current $load_{1}$ of BS *j* belongs to *k*th tier
$\lambda_{j,k}^{load2}$	Current $load_{2}$ of BS *j* belongs to *k*th tier
$P_{j,k}$	Transmission power of a BS *j* belongs to the *k*th tier
$\eta_{i,j,k}$	SINR from BS *j* of tier *k* to UE *i*
$\sigma^{2}$	Noise power level
*W*	Bandwidth
$r_{i,j,k}$	Instantaneous rate from BS *j* of tier *k* to UE *i*
$R_{i,j,k}$	Actual experienced data rate from BS *j* of tier *k* to UE *i*
$x_{i,j,k}$	UE and BS association indicator
$N_{j,k}$	Set of UEs attached to BS *j* of tier *k*
$B_{i}$	Set of BSs the UE *i* can associate with
$U_{i,j,k}\left(.\right)$	Utility of UE *i* from BS *j* of tier *k*
$V_{j,k}\left(.\right)$	Utility of BS *j* of tier *k*
$\Omega_{j,k}$	Set of UEs that the BS *j* of tier *k* can be associated with
$\alpha_{i,j,k}$	Association parameter calculated by UE *i* considering BS *j* of tier *k*
$\delta_{i,j,k}$	Probability of association calculated by UE *i* considering BS *j* of tier *k*

**Table 3:** Comparison of different *d*-choices.

Different Combinations of *d*	Average Jain’s Fairness	Total Number of Probe
*d*1 = 2, *d*2 = 2	0.9985	4
*d*1 = 2, *d*2 = 3	0.9995	5
*d*1 = 2, *d*2 = 4	0.9997	6
*d*1 = 3, *d*2 = 2	0.9985	5
*d*1 = 3, *d*2 = 3	0.9984	6
*d*1 = 3, *d*2 = 4	0.9998	7
*d*1 = 4, *d*2 = 4 (optimal case)	1	14

**Table 4:** Comparison of different algorithms.

Algorithm	Association Rule	Time Complexity	Scheme
1	Centralized	O(U); *U* is the total number of UEs in the network	Greedy based
2	Distributed	O(C); *C* is the average number of BSs a UE can access to	Probability based
3	Distributed	*O* (d1+d2); d1 and d2 are the number of choices in the two levels	d-choices based
